# Total oxidant and antioxidant status and paraoxonase 1 levels ofchildren with noncystic fibrosis bronchiectasis

**DOI:** 10.3906/sag-1503-99

**Published:** 2020-02-13

**Authors:** Ahmet Hakan GEDİK, Erkan ÇAKIR, Aysel VEHAPOĞLU TÜRKMEN, Ömer Faruk ÖZER, Sare Betül KAYGUSUZ

**Affiliations:** 1 Division of Pediatric Pulmonology, Faculty of Medicine, Bezmialem Vakıf University, İstanbul Turkey; 2 Department of Pediatrics, Faculty of Medicine, Bezmialem Vakıf University, İstanbul Turkey; 3 Department of Biochemistry, Faculty of Medicine, Bezmialem Vakıf University, İstanbul Turkey

**Keywords:** Bronchiectasis, noncystic fibrosis bronchiectasis, paraoxonase, total oxidant status, total antioxidant capacity, children

## Abstract

**Background/aim:**

To evaluate total oxidant status (TOS), total antioxidant capacity (TAC), and paraoxonase 1 (PON1) levels in children with noncystic fibrosis (CF) bronchiectasis (BE), and to compare these levels with those of healthy controls. The study parameters were also evaluated according to some demographic, anthropometric, and clinical characteristics, as well as lung functions.

**Materials and methods:**

Enrolled in the study were 118 children with non-CF BE and 68 healthy controls. Serum TOS, TAC, and PON1 levels were determined. Lung function tests were performed by spirometry.

**Results:**

Serum TOS was higher in the patients [median 9.54 (IQR 25–75 = 7.05–13.30) µmol H2O2 Eq/L] than in the healthy subjects [6.64 (5.45–9.53) µmol H2O2 Eq/L] (P < 0.001). TAC was higher in patients with non-CF BE [1.07 (1.0–1.07) mmol Trolox Eq/L] than in the healthy controls [0.87 (0.77–0.98) mmol Trolox Eq/L] (P < 0.001). In addition, serum PON1 levels were significantly higher in the patients [106.5 (42.5–154.2) U/L] than in the controls [47.7 (27.5–82.1) U/L] (P < 0.001). The patients with low FEV1 had decreased TAC when compared to those who had normal FEV1 in non-CF BE.

**Conclusion:**

The present study demonstrated that compared with the control group the children with non-CF BE had elevated oxidative status, antioxidant defenses parameters, and PON1 values.

## 1. Introduction

Bronchiectasis (BE) is a chronic airway disease characterized by abnormal destruction and dilatation of the large airways, bronchi, and bronchioles. Its etiologies include cystic fibrosis (CF), primary ciliary dyskinesia (PCD), genetic abnormalities, immune deficiency syndromes, altered host defense and autoimmune diseases, severe infections, and acquired disorders. The underlying cause of BE may be impossible to identify in about 50% of cases (1). 

In BE, airways are progressively damaged as a result of the interplay between chronic microbial infection, airway inflammation, and tissue-damaging substances secreted by neutrophils, eosinophils, macrophages, and epithelial cells, all of which produce proteinase enzymes and reactive oxygen radicals (2). These radicals interact with various cellular components and macromolecules, and cause metabolic, structural, and functional damage that may lead to cell death (3). During a respiratory burst, excessive quantities of free radicals overwhelm host antioxidant defenses, which then also cause severe damage to the airway epithelial cells and other airway structures (4). When the production of damaging oxidative stress exceeds the capacity of the body’s antioxidant defenses to detoxify it, this condition is known as oxidative stress (5).

Paraoxonase1 (PON1) is one of the important enzymatic antioxidants synthesized by the liver (6). Previous studies have shown that PON1 is an independent risk factor for cardiovascular disease (7); however, the exact antioxidant mechanism of PON1 is not yet known. It is thought that cell damage caused by free oxygen radicals contributes to the pathogens of several chronic respiratory diseases, including asthma, chronic obstructive lung disease, and CF (3,8–11). Only one study touched on oxidative stress in adult patients with BE (12). 

Studies have separately measured serum concentrations of oxidants and antioxidants in laboratories, but the methods used are time-consuming and require complicated techniques (13). New methods to measure total oxidant status (TOS) and total antioxidant capacity (TAC) are rapid, reliable, and inexpensive (14,15). No study in the literature reports having measured the levels of TOS, TAC, and PON1 in pediatric patients with non-CF BE.

The present study investigated the serum TOS, TAC, and PON1 levels in pediatric patients with non-CF BE and compared them with those of healthy controls. In addition, we evaluated the study parameters according to some demographic, anthropometric, and clinical characteristics, as well as lung function tests. 

## 2. Methods

### 2.1. Study design and patients

The required sample size was 65 participants for each group according to the power analysis (power = 0.8 at α = 0.05). Enrolled in this cross-sectional study were 68 healthy controls to compare with 118 pediatric patients who were followed up with the diagnosis of non-CF BE in the Pediatric Pulmonology Division of Bezmialem Vakıf University Hospital from September 2010 to September 2013. The diagnosis of BE was based on standard criteria. The most specific features are (a) the internal diameter of a bronchus wider than its adjacent pulmonary artery, (b) failure of the bronchi to taper, and (c) visualization of the bronchi in the outer 1–2 cm of the lung field (16). High resolution computed tomography (HRCT) was used in all cases. Patients with CF were excluded from the study by two negative measurements of sweat tests and negative CFTR genetic mutation analysis (1).

A detailed disease history of each participant was obtained from the parents. Each subject underwent a complete physical examination and pulmonary function test. During clinical visits, body height and weight were measured using a standard stadiometer and a digital scale so that age- and sex-specific body mass index (BMI) (kg/m2) percentiles could be calculated. 

Because a greater degree of chronic hypoxia can contribute to greater oxidative stress, patients were excluded who were not, during the month prior to the study, in a clinically stable condition, for example by having had respiratory exacerbation or a recent hospital admission. Exacerbation was defined as deterioration of respiratory symptoms, systemic disturbances and radiographing deterioration, or need for hospitalization. Also excluded were children taking dietary supplements with vitamins or omega 3 fatty acids, or using immune suppressive drugs such as systemic corticosteroids. 

The control group consisted of age- and sex-matched children who had been admitted to Bezmialem Vakıf University pediatric outpatient clinic. None of the healthy controls had malnutrition, immune deficiency, or history of immunosuppressive drug use. 

### 2.2. Lung function testing 

Lung function was measured by spirometry. Measurements were performed using the Spirolab III (Medical International Research, Italy) in patients over 6 years old. Forced expiratory volume in the first second of expiration (FEV1), forced vital capacity (FVC), and FEV1/FVC were measured according to standard criteria (17). These parameters were expressed as percent predicted values for each patient’s age, height, and sex. Lung function values <60% were classified as low, 60%–80% were mild, and >80% were normal. 

### 2.3. Blood sample collection and measurements of TOS, TAC, and PON1 levels 

The blood samples were collected from the participants over 2 months (October–November 2013). The samples were centrifuged at 3000 rpm for 10 min. The resultant serum centrifuge was transferred to Eppendorf tubes, and then immediately stored at –80 °C until analysis. 

The TOS of serum was measured by using a novel automated colorimetric measurement method developed by Erel (Rel Assay Diagnostic, Turkey) (14). The color intensity, which can be measured spectrophotometrically, is related to the total amount of oxidant molecules present in the sample. The results are expressed in terms of micromolar hydrogen peroxide equivalent per liter (µmol H2O2 Equivalent/Liter). 

TAC was measured using a novel automated colorimetric measurement method also developed by Erel (Rel Assay Diagnostic, Turkey) (15). In this method, the change in absorbance at 660 nm is related to the total antioxidant level of the sample. The results are expressed as micromolar Trolox equivalent per liter (µmol Trolox Equivalent/Liter). 

The paroxonase1 enzyme activity was measured by using commercially available kits (Rel Assay Diagnostic, Turkey) (18). Paroxonase1 activity was expressed as U/L serum.

### 2.4. Statistical analysis

SPSS version 15.0 was used for analysis. All data were entered into SPSS and evaluated. The numerical parameters were described with the mean, median, and standard deviation; distributions of the categorical measurements were determined by frequencies and percentages. Due to the high range of dissociations, the Mann–Whitney U test was used to compare the levels of TOS, TAS, and PON1 between the patient and control groups. Multiple comparisons were made using Spearman’s correlation and categorical data were evaluated using the chi-square test. P < 0.05 was accepted as statistically significant. 

### 2.5. Ethic committee approval

The study protocol was carried out in accordance with the Helsinki Declaration as revised in 1989. Signed informed consent forms were obtained from the families of all participants. Ethical approval was obtained from the Bezmialem Vakıf University Local Research Ethics Committee (Ethical approval number: 71306642/050–01–04/95). 

## 3. Results

Enrolled in the study were 118 children with non-CF BE and 68 healthy controls. The age distributions of the patients were 0–24 months (n = 9, 7.6%), 25–74 months (n = 6, 5.1%), and ≥75 months (n = 103, 87.4%). No statistically significant differences arose between the patient and control groups as to age, sex, weight, or body mass index (BMI) (Table 1).

**Table 1:** Comparison of demographical properties in children with non-CF bronchiectasis and controls.

Characteristics		Patients(n = 118)	Controls(n = 68)	P* Value
Sex(n, %)	Female	69 (58.5)	42 (61.8)	P = 0.07	Male	49 (41.5)	26 (38.2)
	Age (month) Mean ± SD (min–max)		127 ± 50 (9–192)		106.9 ± 40.7 (11–192)	P = 0.554
Weight, kg Mean ± SD (min–max)		38.5 ± 14.8 (6–78)	41.5 ± 15.1 (8–81)	P = 0.062
Height, cm Mean ± SD (min–max)		141.5 ± 20.6 (61–171)	143.1 ± 25.1 (68–180)	P = 0.091
BMI, kg /m2 Mean ± SD (min–max)		17.8 ± 3.55 (10.1–31)	17.7 ± 3.3 (9.92–28.7)	P = 0.983

Pearson chi-square test was used for statistical analysis, P < 0.05

**Figure 1:**
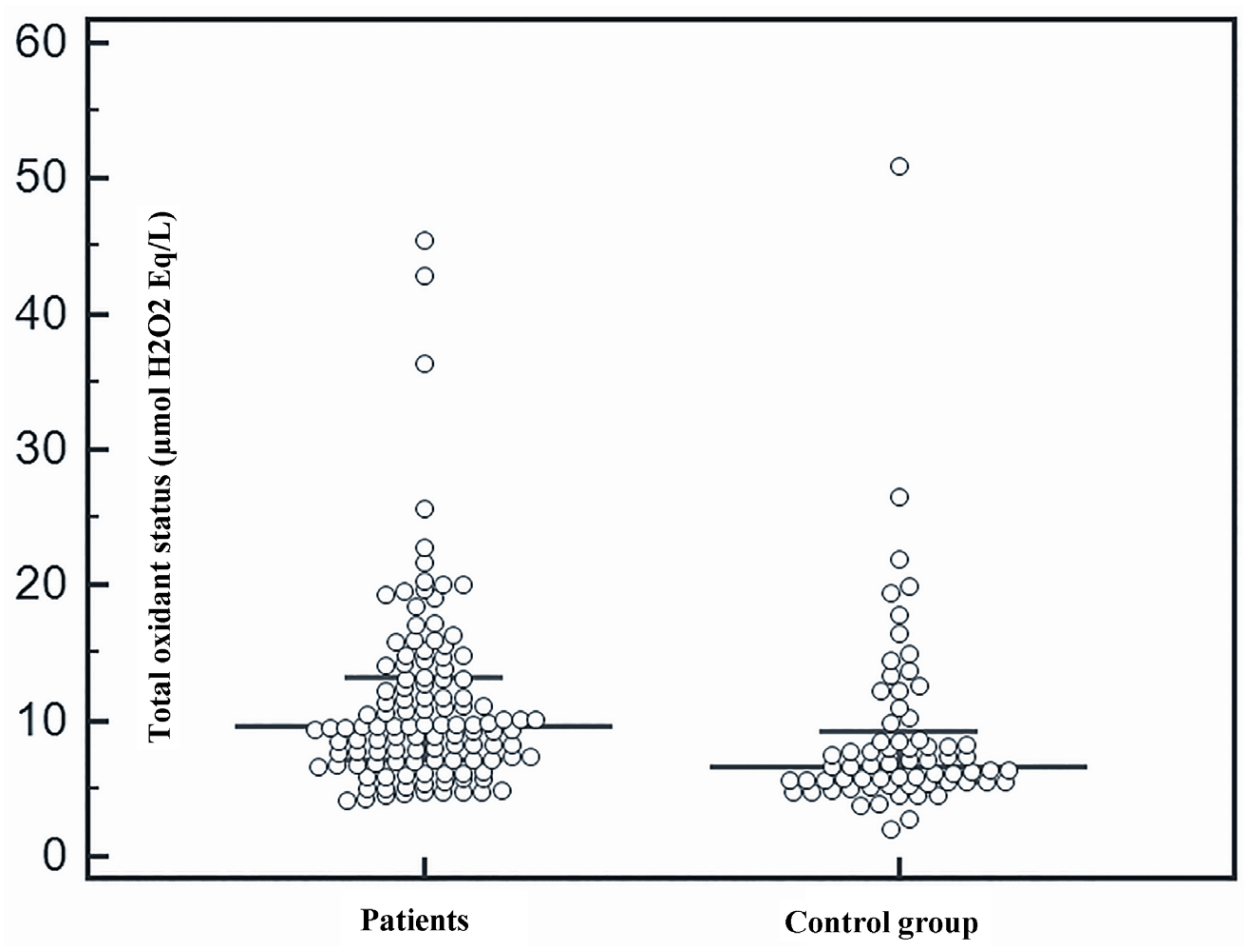
Comparison of total oxidant status between patients and control group.

**Figure 2:**
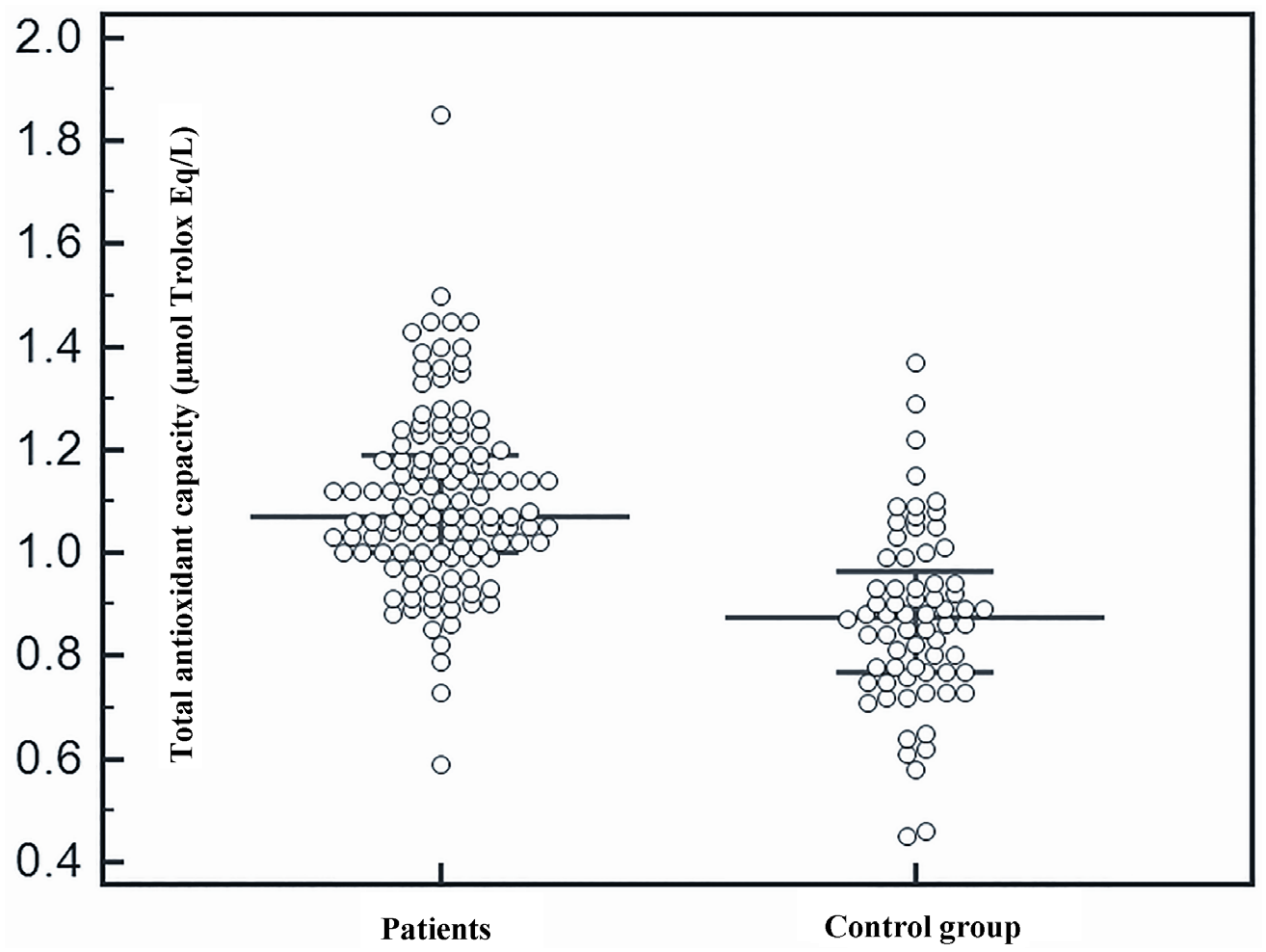
Comparison of total antioxidant capacity between patients and control group.

**Figure 3:**
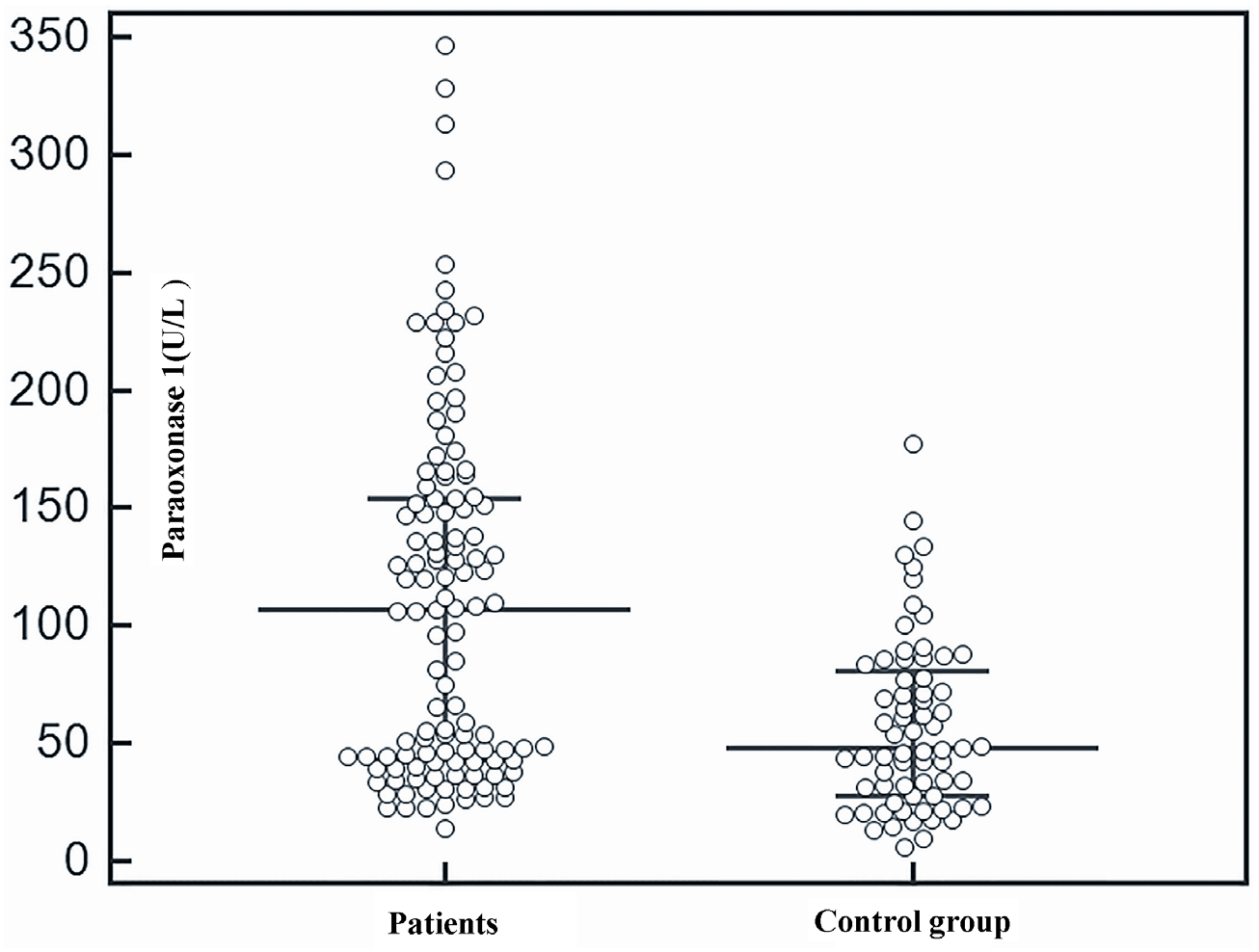
Comparison of paraoxonase 1 levels between patients and control group.

Serum TOS (Figure 1) and TAC (Figure 2) were higher in patients with non-CF BE than in the healthy subjects. In addition, serum PON1 levels were significantly higher among the patients than the controls (Figure 3). See the results of study parameters in Table 2.

**Table 2:** Study parameters of the children with non-CF bronchiectasis and controls.

Parameters	PatientsMedian (IQR 25–75)	ControlsMedian (IQR 25–75)	P value
Total oxidant status (µmol H2O2 Eq/L)	9.54 (7.05–13.30)	6.64 (5.45–9.53)	P < 0.001
Total antioxidant capacity (µmol Trolox Eq/L)	1.07 (1.0–1.07)	0.87 (0.77–0.98)	P < 0.001
Paraoxonase 1 (U/L)	106.5 (42.5–154.2)	47.7 (27.5–82.1)	P < 0.001

Mann–Whitney U test, P < 0.05

No correlation was found between study parameters and demographic and anthropometric characteristics such as age, sex, weight, and BMI (P > 0.05). 

Spirometry was performed in 86 (72.9 %) of the patients in order to examine the relationship between lung function tests and the study parameters. The patients with FEV1 < 60% had decreased TAC compared to those with normal FEV1 (>80%) (P = 0.027). Patients’ FVC and FEV1/FVC ratio showed no statistically significant differences as to TOS, TAS, or PON1 levels (P > 0.05) (Table 3). 

**Table 3:** The study parameters according to lung functions.

Predictedvalues %	FEV1Median (IQR 25–75)			FVCMedian (IQR 25–75)			FEV1/FVCMedian (IQR 25–75)			<80	60–80	<60	<80	60–80	<60	<80	60–80	<60
	n, %	32 (37.2)	29 (33.7)	25 (29.1)	35 (40.7)	32 (37.2)	19 (22.1)	75 (87.2)	11 (12.8)	0
TOS	9.05(6.3–13.1)	9.2(5.9–13.1)	9.7(7.5–11.6)	8.2(5.4–10.6)	11.1(7.0–14.6)	8.8(7.4–10.8)	9.2(6.7–12.5)	9.7(7.4–13.0)	
TAC*	1.04(0.99–1.17)	1.18(1.0–1.4)	1.07(1.0–1.2)	1.1(1.0–1.3)	1.1(1.0–1.2)	1.1(1.0–1.2)	1.1(1.0–1.2)	1.1(1.0–1.4)	
PON1	126.1(44.4–165.6)	65.8(40.9–142.4)	120.2(40.9–170.6)	120.2(44.7–163.7)	95.5(40.5–137.7)	135.9(45.5–195.4)	120.0(42.3–163.7)	96.0(48.7–174.6)	

FEV1 < 60% had decreased TAC compared to those had normal FEV1 (>80%) (P=0.027).

## 4. Discussion

The current study demonstrated that the children with non-CF BE had higher TOS, TAC, and PON1 levels, but no correlation was found between these study parameters and their demographic and anthropometric characteristics. In addition, the patients with low FEV1 had decreased TAC when compared with those who had normal FEV1. 

Chronic pulmonary inflammation and infections increase free radical production. The lungs’ advanced antioxidant system functions to protect them from exposure to harmful oxidants (19). Quantification of oxidative stress can be assessed by the detection of lipid peroxidation end-products derived from the degradation of polyunsaturated fatty acids. Various markers for lipid peroxidation are available and different detection methods have been described in the literature (20). One of them is a novel automated colorimetric measurement method for TOS developed by Erel (14), who also developed another such measurement method for TAC (15). His methodology determines the antioxidative effect of the sample against the potent free radical reactions initiated by the produced hydroxyl radical. To the best of our knowledge, TOS, TAC, and PON1 activity have not previously been investigated in children with BE. 

A lot of research suggests that oxidative stress may contribute to the pathogenesis of many different respiratory diseases, including CF, asthma, pulmonary tuberculosis, and acute respiratory distress syndrome (10,21–23). Lezo et al. demonstrated that the majority of stable CF patients showed elevated oxidative stress markers and plasma antioxidants within the normal range (11). Similarly, Raghunath et al. found that the status of oxidants in plasma levels increased significantly in adult patients who had chronic obstructive pulmonary disease and bronchial asthma (24).

Only one adult study demonstrated that plasma markers of oxidative stress were raised in the adult patients with BE, including those with CF, as compared with the controls, and no differences were seen in the CF patients compared with the other BE patients (25). In our study, TOS and TAC were higher in children with non-CF BE patients. Both chronic inflammation and infections in BE may lead to increased TAC and TOS at the same time. Similarly, some studies found that patients with asthma and chronic tonsillitis had elevated levels of both TAC and TOS (26,27).

Another parameter related to oxidative stress is serum PON1 activity, which could be a key element responsible for oxidative damage in the artery walls of humans. Although some reports have partially investigated this parameter in patients with cardiovascular disease, few studies have evaluated serum PON1 activity in respiratory diseases. Selek et al. showed that PON1 activity was decreased in patients with pulmonary tuberculosis due to reactive oxygen species pathogenesis under conditions of oxidative stress and inflammation (22). Rumora et al. reported that PON1 basal- and salt-stimulated PON1 activity was significantly reduced in patients with chronic obstructive pulmonary disease (28). Acar et al. found that PON1 activity was lower in asthmatics and in COPD than in controls, although no statistically significant difference was found between the two groups (29). Our study found higher PON1 levels. Increased PON1 levels may be due to an impaired antioxidant defense system in BE, which may indicate oxidative stress derived from inflammation. Further studies are needed to clarify the possible mechanisms underlying increased PON1 enzyme activities. 

We have shown that in patients with BE, TOS is not associated with decline in pulmonary parameters; however, the patients with decreased FEV1 had decreased TAC levels compared to those with normal FEV1. This may be explained by the impaired antioxidant capacity relating to an increasing loss of pulmonary function as an indicator of bronchiectasis severity. The limitation of the current study was that the levels of study parameters were detected from the serum of the patients instead of from bronchoalveolar lavage fluid. However, the study was the first study that reflected the levels of those parameters in children with non-CF BE 

In conclusion, this study demonstrates that, compared with the control group, the children with non-CF BE had elevated oxidative status, antioxidant defenses parameters, and PON1 values. In addition, the patients with low FEV1 had decreased TAC. Further studies are needed to understand and explain the mechanisms of these study parameters.

## Acknowledgments

The authors appreciate the contributions and editorial assistance made by S. Delacroix, a native English speaker.
